# Assessment of Volatile Compound Transference through Firefighter Turnout Gear

**DOI:** 10.3390/ijerph19063663

**Published:** 2022-03-19

**Authors:** María José Aliaño-González, Gemma Montalvo, Carmen García-Ruiz, Marta Ferreiro-González, Miguel Palma

**Affiliations:** 1 Department of Analytical Chemistry, Faculty of Sciences, Agrifood Campus of International Excellence (ceiA3), University of Cadiz, The Wine and Food Research Institute IVAGRO, Puerto Real, 11510 Cadiz, Spain; mariajose.aliano@gm.uca.es (M.J.A.-G.); miguel.palma@uca.es (M.P.); 2 Universidad de Alcalá, Departamento de Química Analítica, Química Física e Ingeniería Química, Ctra. Madrid–Barcelona km 33,600, 28871 Madrid, Spain; gemma.montalvo@uah.es (G.M.); carmen.gruiz@uah.es (C.G.-R.); 3 Universidad de Alcalá, Instituto Universitario de Investigación en Ciencias Policiales (IUICP), Calle Libreros 27, 28801 Madrid, Spain

**Keywords:** volatile organic compounds (VOCs), fire, occupational risk, toxicity, turnout gear, firefighter, ion mobility spectrometry, chemometrics, combustion products

## Abstract

There is high concern about the exposure of firefighters to toxic products or carcinogens resulting from combustion during fire interventions. Firefighter turnout gear is designed to protect against immediate fire hazards but not against chemical agents. Additionally, the decontamination of firefighter personal protective equipment remains unresolved. This study evaluated the feasibility of a screening method based on headspace-gas chromatography-ion mobility spectrometry (HS-GC-IMS) in combination with chemometrics (cluster analysis, principal component analysis, and linear discriminant analysis) for the assessment of the transference of volatile compounds through turnout gear. To achieve this, firefighter turnout gears exposed to two different fire scenes (with different combustion materials) were directly analyzed. We obtained a spectral fingerprint for turnout gears that were both exposed and non-exposed to fire scenes. The results showed that (i): the contamination of the turnout gears is different depending on the type of fire loading; and (ii) it is possible to determine if the turnout gear is free of volatile compounds. Based on the latest results, we concluded that HS-GC-IMS can be applied as a screening technique to assess the quality of turnout gear prior to a new fire intervention.

## 1. Introduction

Fire departments are concerned about the contamination of their firefighters’ personal protective equipment (PPE), especially turnout gear, after intervention and how it can affect the firefighters’ health. Contaminants can come from many sources, but they are mainly from incomplete combustion that can generate various substances (soot, volatile organic compounds (VOCs), polycyclic aromatic hydrocarbons (PAHs), or small particles) that can be asphyxiating, irritating, toxic, carcinogenic, or mutagenic [1,2]. In fact, the International Agency for Research on Cancer (IARC) recognizes firefighters within the 18 occupations with a demonstrated increase in cancer cases in relation to the general population [3]. The IARC indicates that some of the contaminants to which this group is exposed are probable carcinogens (Group 2B), and many others, such as several PAHs, 1,3—butadiene, and formaldehyde, are carcinogenic [4,5]. Among the PAHs, benzopyrene is the most common and is classified as a human carcinogen [6]. These aromatic compounds are especially relevant due to their mutagenic and genotoxic properties, and some of them are also considered endocrine disruptors [7,8]. For this reason, there are many studies focused on the assessment of the risk of cancer or mortality in firefighters [5]. Occupational exposure has been associated with an increase in the risk of suffering from several types of cancer (lung, bladder, skin, urinary, and gastrointestinal system), cellular damage due to genetic mutations, oxidative stress, and cardiovascular diseases, which are the first cause of death and the main cause of increased morbidity in firefighters [4,9,10,11,12].

Turnout gear is designed to protect firefighters against immediate fire hazards associated with heat, asphyxiation, and the acute effects of toxic vapors, gases, and particles from the combustion sources. However, it does not offer specific protection (or is not well-characterized) against biological and chemical agents [13,14]. Furthermore, combustion products usually remain impregnated in the coats, other parts of the turnout gear, vehicles, etc., and, therefore, are transferred to the fire station or firefighters’ homes.

Firefighter coats are made with expanded polytetrafluoroethylene (ePTFE), which does not provide specific protection from chemical agents and volatilizes at very high temperatures, releasing toxic products [15]. Consequently, exposure to fire could damage the ePTFE, affecting the coat protection function. If the combustion products remain usually impregnated in the firefighter turnout gear, this creates a microenvironment between the coat and the skin, which may favor the cutaneous absorption of toxic compounds or the transfer of contaminants from clothing to the skin [16,17]. A recent study evaluated chemical vapor diffusion through turnout gear, which was used as a passive membrane. The results showed that chemical vapors, including carcinogens, could diffuse through turnout gear [18].

Coats are normally machine-washed after several firefighting actions, but damage to the ePTFE layer may also occur during the washing-machine-cleaning. However, other sources of contamination may exist between washes, since toxic substances tend to accumulate in the equipment after each intervention, and they can be transferred to a firefighter’s skin, as well as to other people or materials [17,19,20]. Other alternatives, such as the use of ozone chambers, are becoming very popular among fire departments. However, a recent study evaluated the use of ozone chambers as a method to decontaminate PPE [21]. The results of this study showed that after ozone application there was partial, but not total, chemical degradation of PAHs, and new oxygenated PAH compounds that may be even more toxic appeared. Therefore, decontamination is still an unresolved issue, and, so far, there is no method to guarantee the quality of firefighter PPE.

Although some studies have been carried out on the detection of VOCs or, specifically, PAHs in firefighter turnout gear [14], these are very few and are mostly based on the identification of individual compounds using techniques such as liquid chromatography with photodiode array detection [12] or real-time monitoring through the use of specific sensors [22]. In another case, these compounds were determined using wipes that were subsequently analyzed by gas chromatography (GC) [23]. By determining individual compounds, it is possible to identify harmful compounds [24]. However, these methodologies require long analysis time and costly, skilled operators.

Therefore, a screening method capable of determining the level of contamination in PPE in an easy and fast way would be a valuable tool for fire departments to guarantee the cleanness of firefighter coats and to determine when it is necessary to clean them prior to the next usage. In this way, the occupational risk due to harmful combustion products for firefighters will be greatly reduced.

Ion mobility spectrometry (IMS) is an analytical technique based on the mobility of ionized compounds in a gaseous state through a drift tube under a constant electric field at atmospheric pressure [25,26,27]. IMS does not use solvents and does not generate residues, which is a great contribution to the development of green chemistry methods. In addition, it has low detection limits (in the ppb range), it has the ability to monitor analytes in real time, and it works at atmospheric pressure, which greatly facilitates its adaptation to portable equipment. This technique is often combined with other VOC pre-concentration techniques, such as headspace (HS) or separation systems (such as GC) [28,29,30,31] in order to develop fingerprint characteristics of VOCs present in the sample. This technique has already been successfully applied in the chemical detection of warfare agents, agents that cause blisters (mustard gases or lewisite), and asphyxia agents (phosgene) [32,33]. Furthermore, HS-GC-IMS systems have been used in the detection and discrimination of fire debris, allowing the detection of its presence and subsequent characterization, even when it has been exposed to biodegradation processes [34,35,36].

Therefore, the aim of this research is to evaluate the feasibility of the HS-GC-IMS technique as a quick, easy, and reliable screening tool for the assessment of volatile compound transfer through firefighter coats after being exposed to different experiences of indoor fires.

## 2. Materials and Methods

### 2.1. Samples

#### 2.1.1. Firefighters’ Turnout Gears

A firefighter’s coat contains three layers: (i) the outer layer (220 g, 99% aramid and 1% carbon fibre) that allows the firefighter to have greater flexibility and comfort; (ii) the moisture barrier (140 g, ePTFE-laminated aramid) that acts as a waterproof and breathable membrane (that is, permeable to water vapor); and (iii) the internal thermal liner (130 g, 40% aramid, 40% viscose, and 20% Zylon) that acts as a moisture barrier to steam and hot liquids and as protection from bodily fluids and blood-borne pathogens (in the event that a victim needs care). The moisture barrier and the thermal barrier are sewn together as a single piece and attached to the outside using snaps or Velcro.

**Figure 1:**
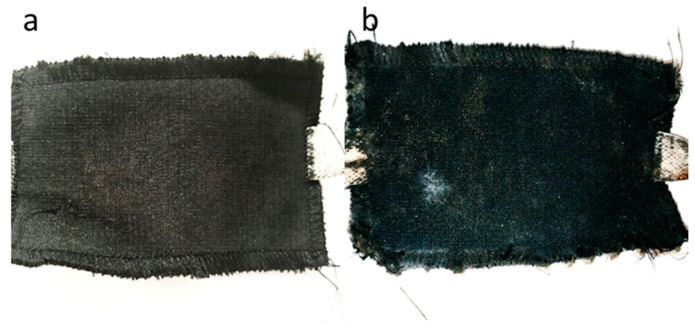
(**a**) Non-exposed armband and (**b**) exposed armband for the assessment of VOC transference on firefighter coats.

For this study, small armbands (10 cm × 5 cm) from new firefighter coats were constructed (i.e., without any type of previous contamination) that were identical to those used in the manufacture of Madrid firefighter personal protective equipment. These armbands were kindly supplied by Protec Solana S.L. (Arnedo, Spain). A cotton rope was sewn to each end to allow a complete fastening, as can be seen in Figure 1. These armbands were placed on the coats of firefighters who participated in activities to extinguish controlled indoor fires. They were positioned 10 cm above the elbow of each firefighter, facing outwards so that they were exposed in the same way a firefighter’s turnout gear would be. Both the placement and subsequent removal were carried out by an analyst, thus avoiding any possible external contamination if it were touched by the firefighter.

Once the exposure of the samples was finished, the armbands were collected and stored in 250 mL metal cans that were perfectly closed, avoiding contamination from outside and avoiding loss of the VOCs. Subsequently, the armbands were cut into 3 square pieces of 3 cm × 3 cm and stored in 10 mL vials, obtaining 3 replicas from each armband. Samples were stored at 0 °C in order to avoid microorganism proliferation until the analysis.

#### 2.1.2. Fire Scenes

Two types of fire scenes with different combustion materials were carried out, both in a firefighter training center (Centro de Entrenamiento contra Incendios—Ilunion Seguridad) in Brunete (Madrid, Spain).

The first fire scene (FS 1) was carried out inside a completely empty 15 m^2^ metal container, where 50 L of diesel was spilled in the center without the use of any type of substrate. In this case, the armbands were exposed to residues generated by the combustion of only diesel oil without the existence of any other combustible material.

For fire scene 2 (FS 2), a car fire was selected. This experience took place inside a 120 m^2^ container. The ignition point was placed in the middle of the car and several wood pieces were used as support. Inside, there was a total of 150 kg of wood to which 5 L of diesel was added. Therefore, one would expect to find volatile compounds and small particles from diesel and wood residues, in addition to residues from the car’s components.

Both fire experiences were repeated for 3 consecutive days. In each fire experience, one armband was tied to each of the three firefighters’ coats, ensuring their total exposure to the fire. Two samples (3 cm × 3 cm) were taken from each armband, located at different positions within it. In addition, 8 samples (3 cm × 3 cm) from new and clean armbands were used as a reference for non-exposed armbands. Samples of armbands not exposed to the fire were named blank. A total of 44 samples were obtained and analyzed in this study (8 blanks + 18 exposed armband samples (3 days × 3 firefighter coats × 2 samples) × 2 fire experiences). On the other hand, the samples exposed to the fires were named FS 1 or FS 2 (depending on whether they were exposed to fire scene 1 or fire scene 2, respectively), followed by the day of exposure (D1, D2, or D3), the sample number (1–3), and _A or _B to distinguish between two replicas of the same armband.

### 2.2. HS-GC-IMS Analysis

All the armband samples were placed into 10-mL glass vials (G.A.S., Dortmund, Germany) and directly analyzed by a FlavourSpec HS-GC-IMS system (G.A.S., Dortmund, Germany) without any pre-treatment. The conditions of the analysis were selected according to the previous experience of the group on fire debris analysis using HS-GC-IMS [37].

### 2.3. Data Treatment

#### 2.3.1. Ion Mobility Sum Spectra (IMSS)

The results from HS-GC-IMS were processed as previously reported for transformation to Ion Mobility Sum Spectra (IMSS), which has produced very good results in the detection and discrimination of fire debris and PDP samples [34,35,36,37]. Once the data matrix was obtained, the drift times were normalized to the reaction ion peak (RIP) with LAV software (G.A.S., Dortmund, Germany), using the ionized water signal as the signal of reference. Subsequently, the IMSS were obtained using LAV software. During this study, the spectra employed contained a total of 1020 drift times in the range of 1.030–2.050 (RIP relative). Spectra were normalized by assigning 1 to the maximum signal in each spectrum. IMSS were arranged in data matrixes named D*mxn*, where m is the number of armband samples and n is the number of drift times.

#### 2.3.2. Chemometric Analysis

Non-supervised techniques, such as hierarchical cluster analysis (HCA) and principal component analysis (PCA), were employed to study the tendency of the samples to be grouped. The supervised technique of linear discriminant analysis (LDA) was used to determine the capacity of the IMS results to discriminate among the three types of samples, i.e., the non-exposed armbands and the ones exposed to FS 1 and FS 2.

The statistical software used for all data treatment were LAV software (G.A.S., Dortmund, Germany) for obtaining IMSS, IBM SPSS Statistics 22 (Armonk, NY, USA) for PCA and LDA, and RStudio software (R version 4.0.5, Boston, MA, USA) for HCA.

## 3. Results and Discussion

### 3.1. Method Development

As previously mentioned, a total of 44 samples were collected for this study: 8 blanks and 36 exposed armband samples (18 samples × 2 fire scenes). The first step was to evaluate the suitability of HS-GC-IMS for detecting differences between the exposed and non-exposed armbands based on the global volatile profiles.

**Figure 2:**
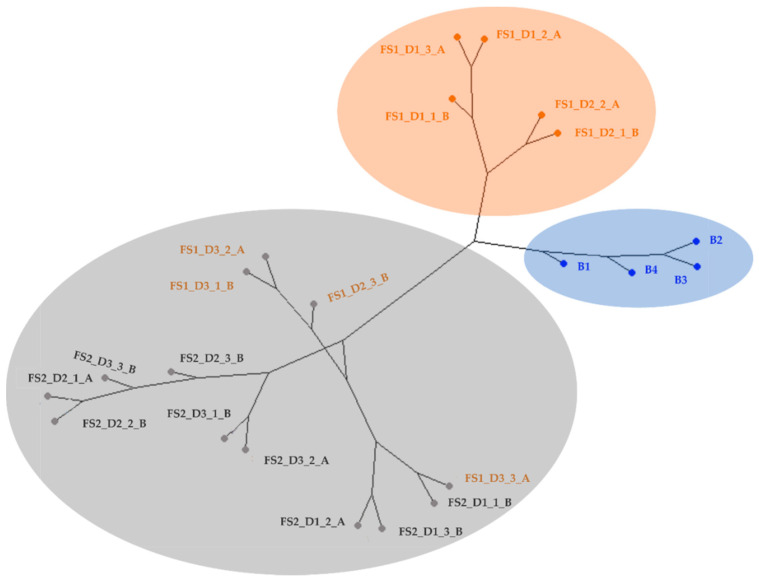
Dendrogram of HCA of blank (blue) and exposed armbands from FS 1 (orange) and FS 2 (grey) (D_22X1020_), where 22 is the number of armband samples, and 1020 is the number of drift times.

The resulting average IMSS of blank armbands and of those exposed to the two fire scenes were analyzed. For this part of the study, 22 samples were analyzed: 4 blank samples and 18 armband samples (all were randomly chosen from the 18 samples from each fire scene). A chemometric study was carried out to prepare a model that categorized the contaminated armbands vs. the blank samples. For that, the 22 IMSS obtained were subjected to HCA. The methodology employed was Ward’s method with squared Euclidean distance. The HCA results are graphically represented in a dendrogram in Figure 2.

As can be observed in Figure 2, blank samples were grouped together (blue dots) and fully separated from the contaminated armbands. These results confirmed that the studied fire scenes produced VOCs that remained in armbands, producing differences in signals than non-exposed armbands. The second cluster (orange dots) grouped five of the nine samples of armbands from FS 1. Finally, the third cluster was divided into two sub-clusters. The first one was formed by the rest of the samples from FS 1 and the samples from FS 2 from day one. The second sub-cluster grouped together the rest of the armband samples from FS 2 (grey dots). To sum it up, the second conclusion obtained from the HCA results was that fire scenes with different characteristics (presence and nature of supports, ignitable liquid, time of burning, etc.) produced different VOCs, and, consequently, different signals that could be employed to determine these characteristics.

**Figure 3:**
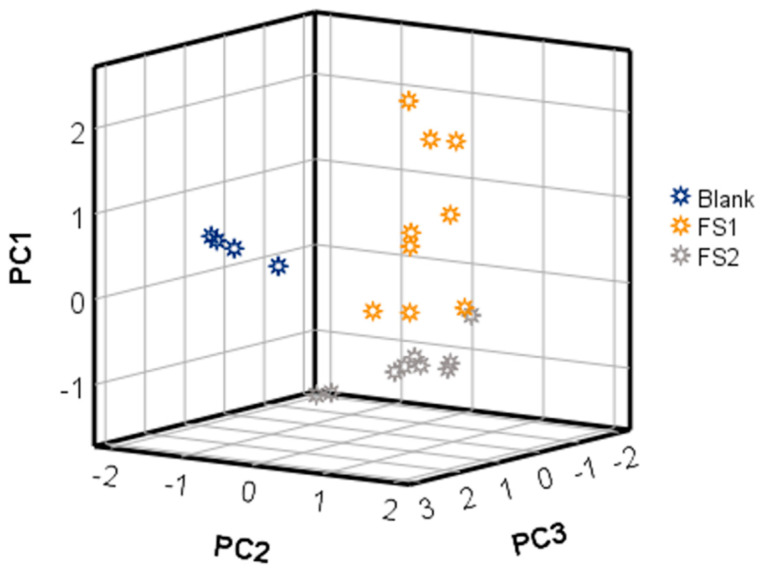
Graphical representation of samples according to the scores from principal components (PCs) 1 to 3 obtained from PCA (D_22X1020_), where 22 is the number of armband samples, and 1020 is the number of drift times.

A PCA was later performed using the same data matrix. Figure 3 displays the samples according to the first three principal components (PCs). The results showed that the first PC achieved 42.5% variance, whereas 89.9% was obtained with the first three PCs (PC1 + PC2 + PC3). As can be observed in Figure 3, PC2 is the main variable responsible for the discrimination, according to exposure or non-exposure armbands. Consequently, these signals could be employed to detect a firefighter coat that can be exposed to a fire scene. On the other hand, armbands from FS 1 and FS 2 were mainly separated in the space according to PC1, which means the drift times that contribute to this principal component could be used to discriminate between fire scenes with and without supports.

These results indicated that the data from HS-GC-IMS were related to the compounds responsible for the discrimination of the armbands according to the presence or absence and type of combustion products, even though the 1020 signals obtained from the IMSS were not sufficient to obtain perfect separation of all analyzed samples.

Based on the tendency shown from the unsupervised analysis, finally, an LDA was carried out using the same data matrix (D_22X1020_) in order to obtain a model for the discrimination of armbands based on VOCs. Three groups were considered a priori: blanks (non-exposed armbands) and armbands taken from FS 1 and FS 2. Cross-validation with stepwise methodology was applied. Table 1 shows Fisher’s linear discriminant functions obtained by LDA that were used to prove the suitability of the method.

A 100% classification was obtained. HS-GC-IMS results showed clear differences in signals caused by VOCs present on firefighter coats, not only between the non-exposed and exposed armbands, but also according to the type of fire scene.

**Figure 4:**
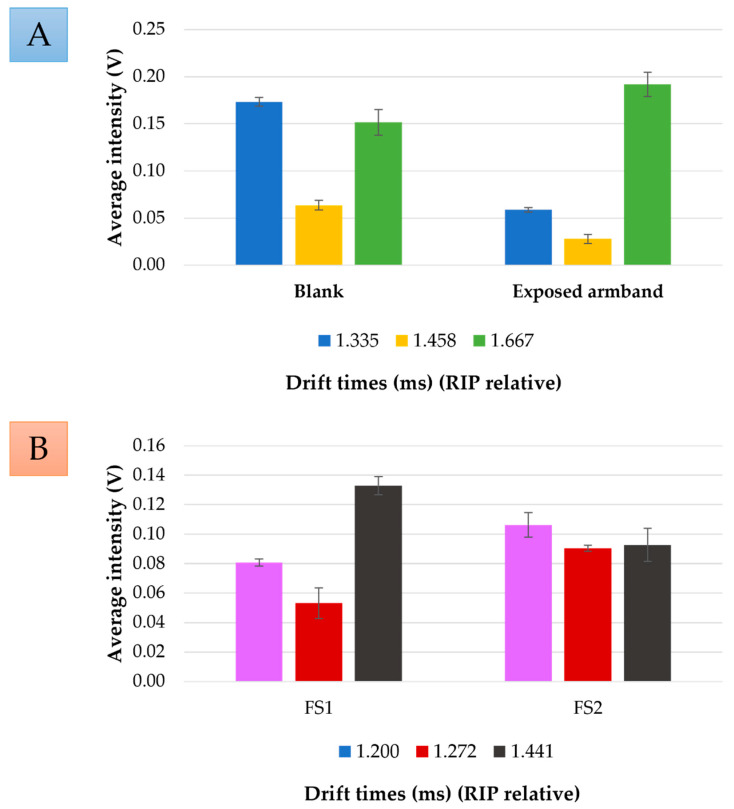
(**A**) Fingerprint obtained from the blank and exposed armbands signals based on the drift times obtained from LDA. (**B**) Fingerprint obtained from the exposed armbands to different fire scenes signals based on the drift times obtained from LDA.

LDA provided different drift times (1.200, 1.272, 1.335, 1.441, 1.458, and 1.667 ms (RIP relative)) important to obtaining the discrimination. By plotting the intensities at these selected drift times, characteristic fingerprints for a non-exposed and an exposed armband, as well as for armband samples of FS 1 and FS 2 were obtained (Figure 4). Figure 4A depicts the average intensity of non-exposed armbands vs. exposed armbands (samples from FS 1 and FS 2). As can be observed, non-exposed armbands exhibited their maximum intensity at 1.335 ms (RIP relative), and exposed armbands showed around 30% of this signal, followed by 1.458 ms (RIP relative) when exposed armbands exhibited around 45% of the signal showed by blanks. On the other hand, the highest intensity from exposed armbands was achieved at 1.667 ms (RIP relative), but non-exposed armbands showed just 80% of the total signal. It was concluded that, with the developed method, only three signals are required to determine whether the firefighter coat was exposed to a fire scene and if VOCs from fire combustion were transferred to the coat.

Moreover, the intensities from samples of FS 1 and FS 2 at the drift times were selected as obtained from LDA for the fire discriminations. Average intensities from samples of FS 1 and FS 2 were calculated and normalized to the maximum intensity of the range. The resulting fingerprints are represented in Figure 4B.

In this case, samples from FS 2 displayed higher intensity than samples from FS 1 at 1.200 and 1.272 ms (RIP relative). Intensities from FS 1 at these times were less than 80% of FS 2 intensities. On the contrary, at 1.441 ms (RIP relative) samples from FS 1 displayed their maximum intensities, whereas samples from FS 2 were just 70% of the FS 1 intensities. Consequently, although more fire scenes should be evaluated, the results discussed above indicate that it is possible to discriminate between fires with different combustion materials using only a few of the signals obtained by HS-GC-IMS analysis without the need of identifying individual compounds.

### 3.2. Validation Study

After testing the suitability of the selected technique for the detection of exposed armbands and the discrimination between armbands from different fires, the next step was to evaluate the applicability of the developed method. For that, the remaining samples (4 blanks and 18 exposed armbands from both fire scenes) that were not employed for model development were analyzed and used for the validation of the model by applying the discrimination functions (Table 1) obtained by LDA. Samples from non-exposed and exposed armbands were successfully discriminated, as well as samples from fire scenes 1 and 2.

These results are in consonance with those observed by Shinde and Ormond [38], who optimized an HS sampling method coupled with GC-MS for the analysis of contaminants on firefighter turnout materials. So far, most of the methods used for the characterization of contaminants in firefighter PPE have been based on the identification of target compound, such as PAHs [14,22,23,24], making this task complex and difficult to reproduce.

In this case, the aim was not to identify individual compounds by HS-GC-IMS, but to study the feasibility of this technique to obtain an overall fingerprint of the odor for determining the global contamination profile (fingerprint) of firefighter equipment. In this case, two different types of fire scenes were studied, and, although a larger variety of fires should be carried out, the obtained results indicated that this technique in combination with chemometrics can be used to develop models for the characterization of samples containing different levels of contaminants. By using pattern recognition techniques, such as LDA, the models “learn” as they analyze more samples, so models are always up-to-date. Therefore, HS-GC-IMS is a viable tool that can be used by firefighter units as a screening method to assess the level of contamination in their equipment.

## 4. Conclusions

The suitability of HS-GC-IMS as a screening method for the detection of volatile compounds in firefighter turnout gear exposed to a fire was evaluated. The results demonstrated that IMSS combined with chemometric techniques, especially with LDA (100% correct classification), can be used for the recognition of non-exposed and exposed turnout gear, even with turnout gear from two different types of fires. The results showed that volatile compounds from the different fire scenes were not the same. Additionally, based on LDA, by using a few drift times (1.200, 1.272, 1.335, 1.441, 1.458, and 1.667 ms), a characteristic fingerprint for an easy and fast identification of the different types of samples was obtained.

In further studies, it would be interesting to study the suitability of the developed method and to characterize the volatile compounds from different fires in order to establish fingerprints according to the contaminants. In this way, it will be possible to guarantee the quality of PPE before using it at a new scene and to decide the proper washing method after different types of fires. The many advantages of this technique in comparison to traditional methods (fast, inexpensive, eco-friendly, easy to use, and portable) make HS-GC-IMS a promising, convenient tool for use in fire departments to reduce the occupational risk of firefighters. In addition, a database with the different fingerprints could be created, and if these fingerprints were correlated with different health risks, it could be possible to create a web platform or application with the generated models in order to automatically evaluate the occupational risk derived from exposure to a fire scene.

## Figures and Tables

**Table 1:** Fisher’s linear discriminant functions obtained from LDA at different drift times.

Drift Time (RIP Relative)/Group	Non-Exposed	FS 1	FS 2
1.200	6806.43	2855.84	2471.55
1.272	4770.23	481.89	1696.80
1.335	43,898.86	11,820.60	13,790.42
1.441	19,788.63	7004.15	6294.30
1.458	−31,983.88	−7892.60	−9503.70
1.667	−5272.90	125.29	−1160.12
Constant	−3034.61	−455.93	−421.54

## Data Availability

Not applicable.
